# The “Mechano-Metabolic-Immune” crosstalk within the skeletal muscle microenvironment: evolution of homeostatic remodeling and quality control mechanisms

**DOI:** 10.3389/fimmu.2026.1875468

**Published:** 2026-07-14

**Authors:** Manli Yan, Xin Zhang, Zhixi Zhu, Hao Liu, Jincheng Zeng, Hua Wei, Lei Huang, Xiang Li

**Affiliations:** 1 The Second Clinical College, Guangzhou University of Chinese Medicine, Guangzhou, Guangdong, China; 2 Department of Endocrinology, Guangdong Provincial Hospital of Chinese Medicine, Guangzhou, Guangdong, China; 3 Department of Operating Rooms, Guangdong Provincial Hospital of Chinese Medicine, Guangzhou, Guangdong, China; 4 Department of Orthopaedic Teaching and Research, Guangdong Provincial Hospital of Chinese Medicine, Guangzhou, Guangdong, China

**Keywords:** immunometabolism, lipotoxicity, mechanotransduction, mitochondrial homeostasis, precision exercise intervention, skeletal muscle quality

## Abstract

Skeletal muscle functions not only as a mechanical apparatus for locomotion but also serves as a pivotal metabolic hub and endocrine organ essential for systemic homeostasis. While traditional perspectives focused on macro-volumetric measurements, contemporary biology posits that muscle quality is fundamentally an integration of mechanotransduction, biochemical metabolism, and ultrastructural coupling. Under comorbidity conditions, the progressive decline of skeletal muscle is intricately linked to multi-systemic dysfunction. In chronic inflammatory environments, mechanical imbalance and metabolic derangements are not merely additive; instead, they construct a sophisticated “mechano-metabolic-immune” network by co-regulating immune cell phenotypes and inflammatory thresholds. Pathological remodeling represents the destabilization of this homeostatic axis: lipotoxic metabolic stress induces the phenotypic deviation of fibro-adipogenic progenitors (FAPs) and M1 polarization of macrophages, establishing a pro-inflammatory priming state. Furthermore, the leakage of mitochondrial DNA (mtDNA) resulting from impaired mitochondrial quality control amplifies local metabolic disturbances into cGAS-STING pathway activation that secondary drives macrophage M1 polarization, serving as a critical driver of muscle atrophy. Within this pathological context, mechanical signals act not only as physical stimuli but as active variables that remodel microenvironmental stability. Through molecular transducers such as Piezo1, FAK, and TRPV4, kinetic loading facilitates mechano-chemical transduction and activates the energy sensor AMPK, thereby maintaining mitochondrial dynamic equilibrium and suppressing inflammatory cascades. This metabolic remodeling promotes the transition of macrophages toward a pro-regenerative/anti-inflammatory phenotype, supporting functional maintenance by resolving chronic inflammation and restoring tissue homeostasis. This review proposes the “mechano-metabolic-immune” axis as a pivotal regulatory framework governing skeletal muscle quality. Given that the biological benefits of mechanical intervention are constrained by physiological thresholds, precisely defining exercise load parameters across diverse pathological backgrounds is a rational foundation for transitioning from macro-rehabilitation to mechanism-driven precision interventions targeting FAPs adipogenic differentiation, intramuscular fat accumulation, and AMPK-mediated mitochondrial quality control, providing essential criteria for developing safe and effective clinical exercise prescriptions.

## 1 Introduction

Skeletal muscle constitutes approximately 40% to 50% of the total body mass in healthy individuals. Beyond its role as a mechanical apparatus driving locomotion and thermogenesis, it functions as a central node for maintaining systemic metabolic homeostasis (1). Substantial evidence has demonstrated that skeletal muscle exhibits remarkable biological plasticity, enabling it to respond to dynamic challenges from functional loading, nutritional metabolism, and genetic instructions through multifaceted pathways (2–6). However, traditional perspectives have long restricted the evaluation of skeletal muscle quality to macro-linear measurements, such as muscle volume or fiber diameter (7). This unidimensional definition is no longer sufficient to encompass the complex connotations established by contemporary biology. In fact, muscle quality has evolved into a multidimensional systemic attribute characterized by the deep coupling of mechanotransduction, biochemical metabolism, and ultrastructural integrity.

In the contemporary clinical landscape, multimorbidity has emerged as a primary threat to skeletal muscle homeostasis, as the coexistence of multiple chronic conditions is intricately linked to the progressive decline of muscle quality and function (8–10). Systemic stressors induced by obesity (11), metabolic disorders (12, 13), respiratory (14–16) and cardiovascular diseases (17, 18), synergistically drive the pathological remodeling of skeletal muscle. Within this context, osteoarthritis serves as a quintessential cross-tissue comorbidity model that integrates biomechanical imbalance with sterile inflammation. The joint pain and structural degeneration inherent to osteoarthritis profoundly alter the microenvironment of adjacent skeletal muscles, primarily by inducing protective unloading (mechanical unloading) and the spillover of localized inflammation into neighboring tissues (19, 20). This pathological cross-talk between the joint and muscle not only accelerates skeletal muscle degeneration, but the secondary muscle weakness conversely exacerbates joint instability, thereby establishing a distinct musculoskeletal vicious cycle within the framework of comorbidities.

**Figure 1:**
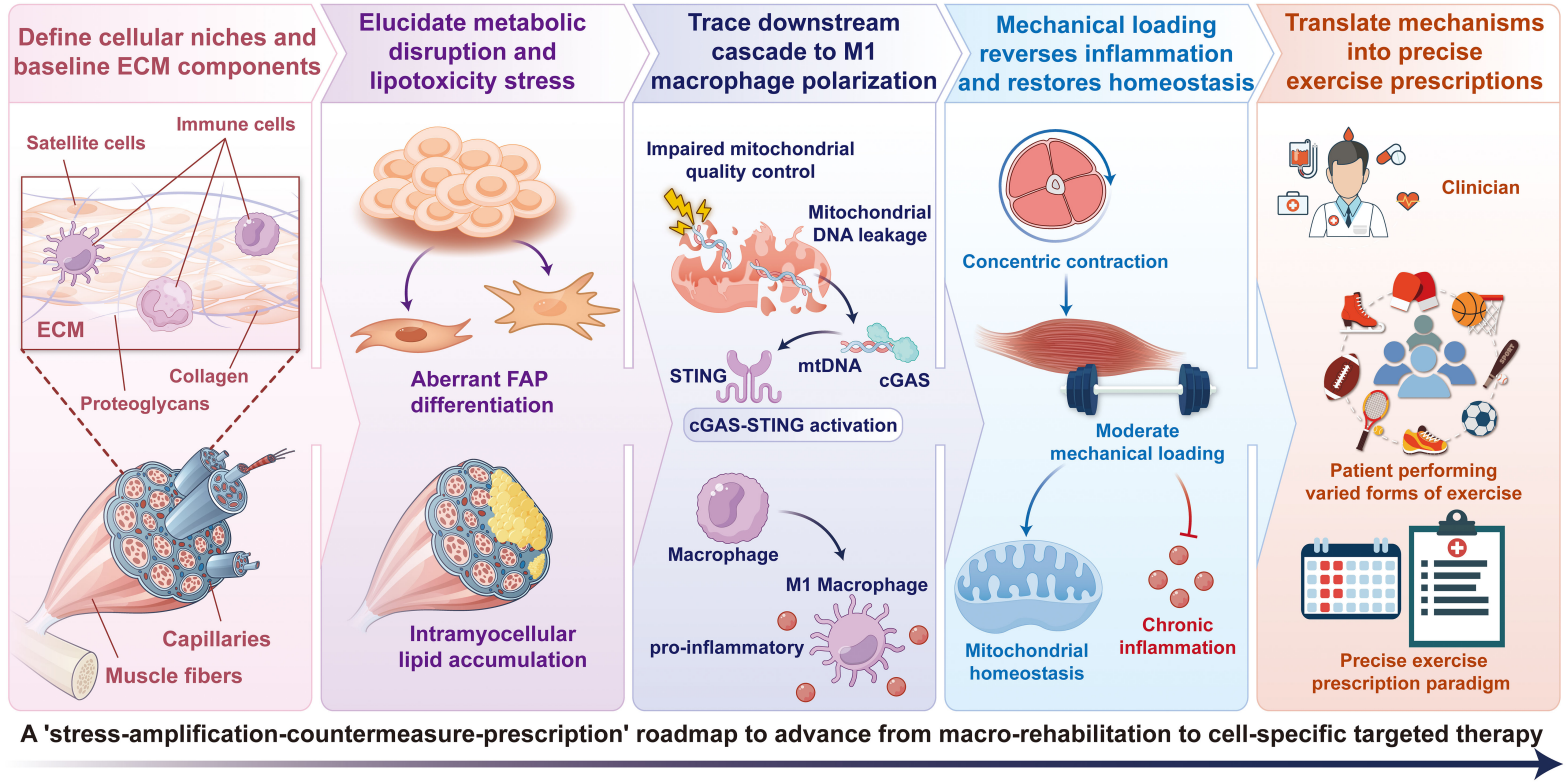
A mechanistic roadmap transitioning from metabolic-immune stress to precise exercise prescriptions in skeletal muscle.

Diverse pathological mechanisms synergistically drive the process of skeletal muscle pathological remodeling under the conditions of comorbidities. However, despite in-depth investigations in previous literature regarding isolated domains—such as mechanical signal transduction, metabolic homeostasis imbalance, or localized immune activation—the cross-scale integration of physical dynamic loading and intracellular metabolic sensing within a specific immune microenvironmental context remains poorly understood. Within the intricate clinical landscape of comorbidities, the mechanical, metabolic, and immune dimensions do not exist in isolation, nor do they represent a simple superposition of effects; rather, they jointly dictate the fate of skeletal muscle through highly dynamic cross-talk (21–25). Currently, a systematic theoretical framework elucidating this tripartite regulatory hub is still lacking in the academic community (**Figure 1**), which substantially impedes a profound understanding of the evolutionary mechanisms underlying musculoskeletal degenerative diseases. Consequently, this review focuses on the specific pathological contexts of chronic metabolic diseases (e.g., obesity, type 2 diabetes) and their associated musculoskeletal comorbidities (e.g., osteoarthritis, diabetic myopathy). It systematically explores the highly interactive “mechanics-metabolism-immunity” remodeling mechanisms within these comorbidity networks, aiming to establish a systematic theoretical foundation for subsequent precision intervention studies in this field.

## 2 Metabolic stress-induced inflammation and skeletal muscle quality remodeling

### 2.1 Multidimensional composition of the skeletal muscle microenvironment

The physiological functions of over 600 skeletal muscles in the human body are highly dependent on their sophisticated microenvironmental composition. The distribution ratio and phenotypic plasticity of myofibers—ranging from slow-twitch oxidative to fast-twitch glycolytic types—establish the foundational balance between energy utilization and mechanical output (26). Concurrently, the dynamic interaction between stromal/progenitor cells, such as fibro-adipogenic progenitors (FAPs), and infiltrating immune cells constitutes the core cellular network regulating tissue regeneration and chronic inflammatory phenotypes (27, 28); intramuscular adipose infiltration disrupts local homeostasis and triggers histopathological remodeling through physical displacement and paracrine cascades of pro-inflammatory cytokines (29–31). Meanwhile, the evolution of extracellular matrix (ECM) stiffness serves as a critical physical signal transduction medium, regulating the activation potential of muscle satellite cells and the differentiation fate of fibroblasts (32–34). Meanwhile, the structural integrity of the microvascular-neuromuscular axis is essential for sustaining localized blood perfusion and neural innervation within the muscle (35–37). At the cellular metabolic level, derangements in mitochondrial dynamics and the failure of quality control systems not only induce neuromuscular junction dysfunction and denervation-like changes via energy depletion but also directly mediate the early decline of muscle strength through oxidative stress products (38, 39). This multidimensional system defines more than just contractile efficiency; it profoundly reveals the pathophysiological essence of skeletal muscle as the body’s largest endocrine organ and metabolic reservoir, establishing its pivotal role in the precision regulation of immune responses (40).

### 2.2 Lipotoxic microenvironment and inflammatory cascades: metabolic stress-driven tissue remodeling

#### 2.2.1 Cellular homeostasis imbalance: phenotypic deviation of FAPs and intramuscular adipose infiltration

Within the chronic metabolic stress networks established by comorbidities, the destabilization of the skeletal muscle microenvironment often originates from phenotypic abnormalities at the cellular level (41–43). As a core, non-myogenic component residing in the interstitial spaces between muscles, FAPs possess a high capacity for regulating microenvironmental homeostasis under physiological conditions (44). FAPs sensitively integrate immune responses with stem cell activity (45), providing essential microenvironmental support for muscle repair by releasing paracrine factors and remodeling the extracellular matrix (46). However, in the context of aging or chronic disease, a persistent inflammatory environment can induce a phenotypic deviation in FAPs. This shift transforms them from repair supporters into pro-fibrotic and adipogenic cells, directly leading to significant fibrosis and ectopic lipid deposition (i.e., myosteatosis), which subsequently impairs contractile function and increases the risk of re-injury (46, 47).

The accumulation of intramuscular adipose tissue (IMAT) is a critical factor leading to the loss of skeletal muscle strength and physical function (48, 49). In elderly populations, myosteatosis significantly slows the rate of torque development and reduces peak torque in the quadriceps; notably, this impairment of functional output occurs independently of the loss of muscle mass (50). In patients with obesity complicated by diabetes or peripheral neuropathy, excessive infiltration of leg fat is closely associated with diminished calf muscle strength and impaired overall motor function (51), exhibiting distinct quality-driven characteristics. Consequently, the abnormal accumulation of IMAT not only reflects pathological alterations in tissue composition but also serves as a core predictive indicator for the decline of skeletal muscle function.

Ectopic lipid deposition is not metabolically inert; instead, it exhibits a significant pro-inflammatory phenotypic transition. Hypertrophic adipocytes trigger local and systemic chronic low-grade inflammation by recruiting macrophages and secreting adipokines such as leptin, resistin, and tumor necrosis factor-alpha (TNF-α) (52, 53). This paracrine effect intertwines with myokines and osteokines to construct a complex endocrine regulatory network, which impairs the contractile function of adjacent myofibers and interferes with musculoskeletal metabolic coupling, serving as a critical factor driving the pathological progression of various chronic diseases (53).

#### 2.2.2 Molecular mechanisms: from lipotoxic intermediate accumulation to impaired insulin signaling

Within the skeletal muscle microenvironment, the excessive delivery and uptake of fatty acids (FA) are critical factors inducing metabolic dysfunction. When intracellular FA entry exceeds the capacity for oxidative load, FAs are first activated into fatty acyl-CoA. Subsequently, depending on the metabolic flux, they are either sequestered as triacylglycerols within lipid droplets or further converted into lipotoxic intermediates, such as diacylglycerols and ceramides (54). The accumulation of these lipotoxic molecules directly mediates the disruption of metabolic homeostasis by interfering with the insulin signaling cascade.

Research has demonstrated that an increase in intracellular fatty acyl-CoA is associated with a significant reduction in insulin-induced IRS-1 tyrosine phosphorylation and associated PI3K activity by approximately 30% and 50%, respectively, while the phosphorylation levels of the insulin receptor itself remain stable (55). The core mechanism of this inhibitory process lies in the activation of the serine kinase PKC-θ (55, 56), which mediates increased phosphorylation at the IRS-1 Ser307 site. This modification creates steric hindrance, thereby blocking the canonical signaling pathway. Furthermore, as key lipotoxic products, the levels of ceramides and diacylglycerols are closely correlated with insulin sensitivity; modulating their metabolic flux can effectively alleviate metabolic impairment in skeletal muscle (54, 57–59).

Crucially, lipotoxic intermediates function as endogenous stress signals that activate the c-Jun N-terminal kinase (JNK) and inhibitor of nuclear factor κB kinase subunit beta (IKKβ) axis (60). This process not only directly impairs insulin signaling by inducing the serine phosphorylation of IRS-1 (61), but also upregulates the expression of pro-inflammatory factors (62). Concurrently, pro-inflammatory cytokines further activate the JNK and IKKβ/NF-κB pathways through canonical receptor mechanisms in a positive feedback loop, establishing a vicious cycle between inflammation and metabolic derangement (63, 64). This fatty acid-initiated molecular inhibition pattern shares high homology with the TNF-α-mediated inflammatory signaling pathway, suggesting an intricate intertwining and co-regulation of metabolic stress and inflammatory responses at the molecular level (55, 60, 62, 63).

#### 2.2.3 Immunoinflammation: the fatty acid-TLR4 axis and M1 macrophage polarization

Macrophages account for approximately 10% of the total immune cell population and play a pivotal role in inflammatory responses and the regulation of tissue homeostasis due to their remarkable heterogeneity and plasticity. Beyond their canonical immune surveillance functions, macrophages are recognized as essential regulatory factors in metabolic diseases. Fatty acids serve not only as energy substrates and structural components but also significantly influence the polarization phenotype and function of macrophages by modulating key signaling pathways (65).

In a lipid-overloaded microenvironment, excessive saturated fatty acids mediate ligands that activate the Toll-like receptor 4 (TLR4) signaling pathway on the macrophage surface. By triggering canonical pro-inflammatory pathways such as the NF-κB cascade, this process induces the expression of pro-inflammatory cytokine precursors, thereby initiating innate immune responses (66, 67). Under environmental stress driven by both metabolites and pro-inflammatory adipokines, infiltrating macrophages polarize toward the M1 (pro-inflammatory) phenotype (68, 69). During this process, cells undergo significant metabolic reprogramming, characterized by a reduction in mitochondrial oxidative phosphorylation accompanied by compensatory increases in glycolytic rates and de novo fatty acid synthesis (70).

Furthermore, the activation of TLR4 exerts multifaceted pathological effects, directly inducing myofibroblast differentiation and enhancing the biosensitivity of fibroblasts to TGF-β (66, 71). As a primary driver of the fibrotic process, the TGF-β/Smad pathway promotes the differentiation of FAPs into myofibroblasts and stimulates extracellular matrix synthesis. Concurrently, YAP/TAZ further amplifies the fibrotic response by integrating mechanical and biochemical signals (72). Within the dual context of mechanical imbalance and metabolic derangement, these effects are further exacerbated, synergistically driving tissue remodeling and the fibrotic progression of skeletal muscle (72, 73).

**Figure 2:**
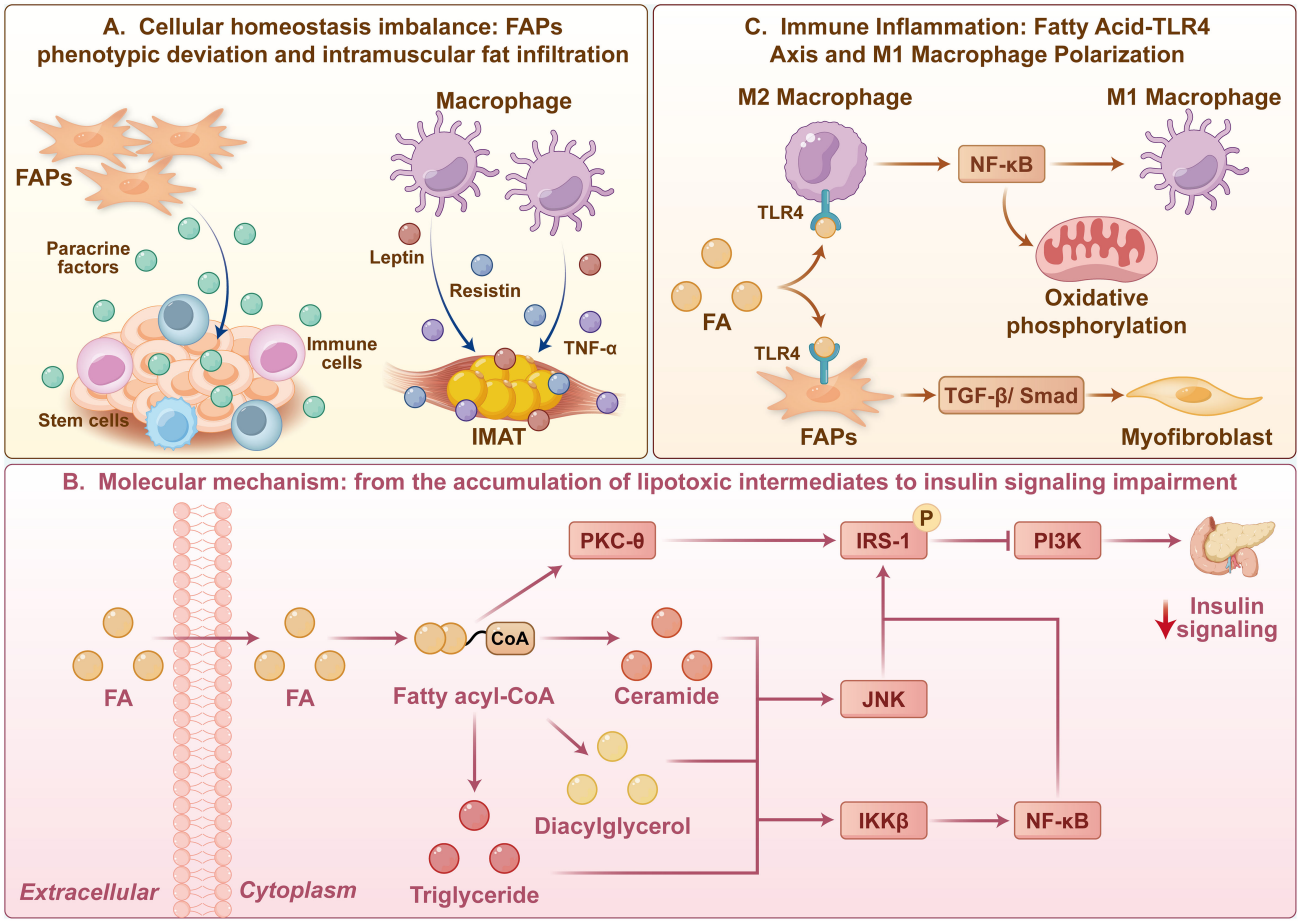
Pathophysiological cascades in the skeletal muscle microenvironment: integration of cellular homeostatic imbalance and lipotoxic signaling.

In summary, the lipotoxic microenvironment induces multidimensional tissue damage by remodeling the immunometabolic landscape of skeletal muscle (**Figure 2**). The diversion of lipid metabolic flux disrupts insulin signaling through steric hindrance effects, while concomitant fatty acid overload initiates the pro-inflammatory polarization and metabolic reprogramming of macrophages via the TLR4 axis. This intricate intertwining of metabolic stress and inflammatory responses at the molecular level constitutes the important pathological basis for skeletal muscle functional decline and fibrotic progression.

### 2.3 Mitochondrial dysfunction and oxidative stress: the intersection of metabolic failure and immune amplification

#### 2.3.1 From metabolic hub to pro-inflammatory source: failure of the mitochondrial quality control system

Mitochondria exist as a dynamic network precisely regulated by biogenesis, fusion, fission, and mitophagy. This highly integrated kinetic process forms the physical foundation for the metabolic flexibility and mechanical homeostasis of skeletal muscle (74–76). Studies have demonstrated a significant imbalance in mitochondrial homeostasis within the skeletal muscle of individuals with obesity and type 2 diabetes, characterized by decreased mitochondrial abundance (e.g., downregulated gene and protein expression, reduced citrate synthase activity) and aberrant morphological structures (77). Notably, systemic regulation by multidimensional factors—including age, body composition, physical activity, and genetic background—results in pronounced pathological heterogeneity in the degree of mitochondrial functional impairment (77–79). This underscores the complex pathological landscape of mitochondrial damage in skeletal muscle within the context of chronic disease.

The dysregulation of coupling between mitochondrial morphology and function, which triggers metabolic remodeling, serves as a central mechanism in skeletal muscle dysfunction. Lipid overload is a critical trigger for the derangement of mitochondrial dynamics. Excessive fatty acid uptake facilitates the phosphorylation of the mitochondrial fission protein Drp1 at the Ser616 site by activating the ERK signaling pathway (80). Combined with the intracellular acetylation microenvironment induced by lipid metabolic flux (81), these factors synergistically drive the pathological fragmentation of mitochondrial networks. This kinetic imbalance directly results in the decay of mitochondrial membrane potential and a reduction in oxygen consumption, which subsequently hinders normal fatty acid oxidation (82), establishing a metabolic vicious cycle. In skeletal muscle models, the knockdown of Drp1 leads to pathological hyperfusion of mitochondria, characterized by decreased fatty acid oxidation, abnormal accumulation of succinate within muscle tissue, and impaired insulin signaling. These findings indicate that Drp1 not only mediates morphological evolution but also plays a pivotal role in maintaining the assembly of mitochondrial respiratory chain complex II, thereby ensuring lipid oxidation efficiency and insulin sensitivity (83).

Within the context of chronic inflammation, the failure of the mitochondrial quality control system further amplifies pathological effects. The accumulation of defective mitochondria resulting from impaired mitophagy not only exacerbates energy metabolic disorders but also profoundly disrupts the calcium homeostasis axis of skeletal muscle by damaging the ultrastructure of calcium release and calcium entry units (74, 84–86). The dysregulation of structural-functional coupling leads to abnormal Ca^2+^ handling and a deficit in ATP supply, which directly weakens the efficiency of excitation-contraction coupling and excitation-metabolism coupling. This serves as a core mechanism mediating the decline in muscle performance and increased susceptibility to fatigue associated with aging and denervation.

More critically, mitochondria serve not only as the core of cellular ATP production but also as dynamic hubs regulating cellular homeostasis and immune responses (87). Chronic infection or persistent inflammation disrupts the endosymbiotic homeostasis between the host and mitochondria, prompting a transition of mitochondria from metabolic hubs to endogenous immune triggers (88, 89). During this process, mitochondria release damage-associated molecular patterns (DAMPs) with distinct bacterially conserved features, such as mitochondrial DNA (mtDNA). The prokaryotic structural characteristics retained by these molecules blur the distinction between exogenous infectious signals and endogenous damage signals, making them key endogenous immunogens that mediate inflammatory responses. Various cellular stressors can compromise mitochondrial integrity, causing mtDNA to escape into the cytoplasm or the extracellular environment. Once released, mtDNA acts as a potent DAMP, transmitting cellular danger signals and initiating intense inflammatory cascades. This process drives a self-sustaining inflammatory feedback loop that exacerbates tissue damage through amplified immune activation; while essential for protective immunity against pathogens, it also underlies the pathogenesis of various sterile inflammatory diseases and malignancies (87–89).

#### 2.3.2 Activation of immune cascades: the role of the cGAS-STING axis in muscle deterioration

Compared to satellite cells, the high contractile activity of muscle fibers relies on highly active mitochondrial metabolism. This elevated metabolic load renders them more susceptible targets for oxidative stress-induced damage (90). Clinical and basic research has confirmed the central role of the cGAS-STING pathway in the pathological remodeling of skeletal muscle. In animal models of premature aging, defects in mitochondrial dynamics and mitophagy-mediated repair lead to the massive accumulation of oxidatively damaged mitochondria, which in turn triggers the loss of mitochondrial membrane integrity and the leakage of mtDNA into the cytoplasm. This process ectopically activates the cGAS-STING pathway, driving chronic inflammation and aging-related phenotypes in skeletal muscle (90, 91).

A study by Zhao et al. further provided clinical evidence: mtDNA loads were significantly elevated in the serum of patients with mitochondrial diseases, and protein levels of cGAS, STING, TBK1, and IRF3 were abnormally upregulated in skeletal muscle biopsies, directly confirming the pathological recruitment of this axis in human muscle tissue (92). Notably, under physiological conditions, the cGAS-STING-NF-κB pathway exerts a compensatory regulatory role, promoting the phenotypic remodeling of oxidative muscle fibers by moderately downregulating the myogenic determination factor MyoD, thereby maintaining the exercise adaptability of skeletal muscle (93). However, under pathological conditions driven by lipotoxicity or chronic stress, the overactivation of this pathway triggers a persistent inflammatory cascade. This not only induces programmed cell death and cellular senescence but also inhibits the activity and regenerative potential of skeletal muscle satellite cells, ultimately mediating muscle atrophy and tissue fibrosis (94).

In pathological models such as diabetic myopathy, the consequence of this immune amplification is directly manifested as the macroscopic deterioration of muscle mass. Research has demonstrated that the deficiency of STING significantly alleviates diabetes-induced muscle atrophy. The underlying mechanism is closely associated with the inhibition of the expression of pyroptosis-related proteins, such as NLRP3, caspase-1, and GSDMD-N, as well as the release of inflammatory chemokines. STING activates the NLRP3 inflammasome through its ion channel activity, driving cells toward programmed pyroptosis, whereas the inhibition of this pathway can significantly curtail this pathological process (95).

From the perspective of translational medicine, this pathological state driven by metabolic derangement and immune amplification exhibits reversible potential upon the introduction of specific mechanical loading: endurance training can inhibit the aging-induced elevation of proteins such as STING, NLRP3, and GSDMD, and reduce cytosolic mtDNA levels, thereby antagonizing skeletal muscle atrophy triggered by chronic inflammation (96). This evidence not only reveals the critical role of the metabolic-immune axis in regulating muscle mass but also highlights the biological effects of mechanical signals—such as exercise training—in remodeling the muscle immune microenvironment and restoring mitochondrial homeostasis (96, 97).

**Figure 3:**
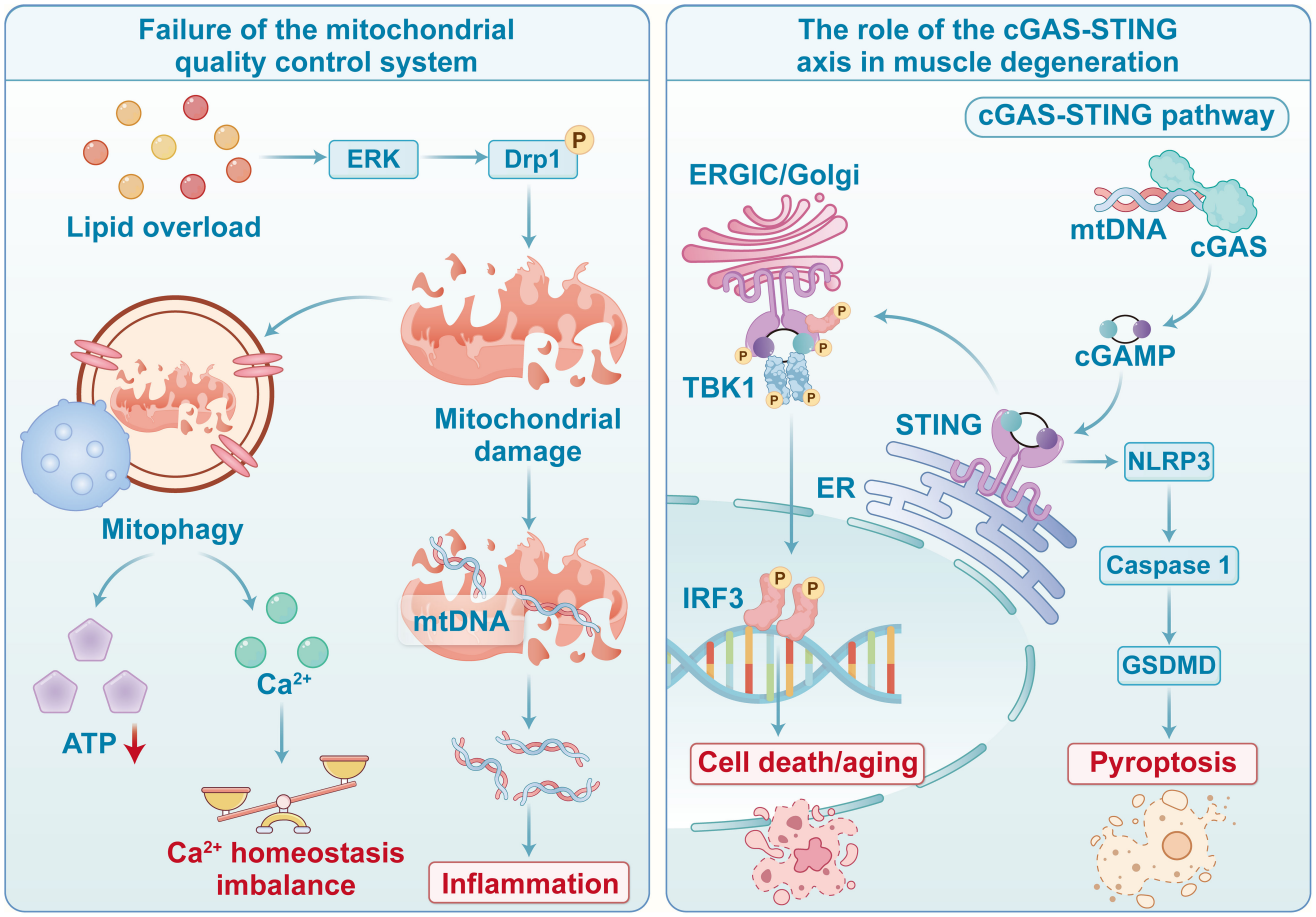
Disruption of mitochondrial homeostasis and activation of the cGAS-STING-NLRP3 signaling cascade during myocyte senescence.

In summary, the activation of the cGAS-STING pathway—triggered by the imbalance in mitochondrial quality control—amplifies skeletal muscle metabolic derangement into systemic inflammation, serving as a critical node that induces the simultaneous decline of muscle mass and function (**Figure 3**). Within this complex pathological network, mechanical loading, as an exogenous interventional variable, demonstrates significant potential in inhibiting mtDNA leakage and downregulating inflammatory cascades. Consequently, elucidating the regulatory mechanisms of mechanical signals on the skeletal muscle immune microenvironment has become a pivotal link bridging fundamental metabolic research and clinical rehabilitative translation.

## 3 Regulation of the muscle immune microenvironment by mechanical signals

### 3.1 Sensing modalities and signal transduction in the muscle mechanical microenvironment

As the largest locomotor organ of the human body, skeletal muscle is perpetually anchored within a dynamic and complex physical stress field. Its sophisticated hierarchical structure and unique mechanical properties endow the tissue with the capacity for precise response to and tolerance of physiological dynamic loading. During physiological functioning, skeletal muscle does not respond to single stimuli in isolation; rather, through complex spatiotemporal superposition, it systematically integrates and transduces four core mechanical modalities: periodic stretching generated by muscle fiber contraction (98), fluid shear stress formed by interstitial fluid flow (99), axial compressive forces under load-bearing conditions (100, 101), and matrix stiffness determined by extracellular matrix remodeling (102, 103). These factors collectively define the characteristics of the skeletal muscle mechanical microenvironment. Cells perceive extracellular stress through the process of mechanotransduction and adjust their metabolic activity in real-time to adapt to the dynamic environment (104, 105). However, when trauma or aging leads to structural disorganization, drastic shifts in stress and strain distribution disrupt the original biomechanical homeostasis, significantly weakening the cells’ ability to perceive and respond to mechanical signals (106).

Among the molecular hubs mediating mechanotransduction, the Piezo family, acting as mechanically activated non-selective cation channels, plays a dominant role (107–109). In the musculoskeletal system, PIEZO1 is primarily responsible for maintaining muscle and bone mass, sensing tendon stretch and tissue injury, and regulating mechanical stimulus responses within cartilage and intervertebral discs. In contrast, PIEZO2 focuses on transmitting pain, touch, and proprioception (109, 110). Within skeletal muscle, PIEZO1 serves as a critical hub regulating the fate determination of muscle satellite cells (MuSCs) (111, 112). The deficiency of PIEZO1 leads to the abnormal premature activation of MuSCs and impairs their differentiation potential. The specific mechanism involves Ca^2+^ upregulation and the activation of the cPKC/NOX4 axis, which in turn triggers ROS accumulation and p53-dependent cellular senescence (111). Furthermore, PIEZO1 maintains muscle regenerative homeostasis by regulating the dynamic transition of MuSCs among three functional states: responsive, intermediate, and sensory. In models of muscular dystrophy, the downregulation of PIEZO1 activity is a key factor leading to regenerative defects, whereas the pharmacological activation of PIEZO1 can effectively improve the disease phenotype (112).

At the level of pathological mechanisms, abnormal mechanical stress triggers a PIEZO1-mediated inflammatory and fibrotic cascade. Research indicates that pathological stress overload can aberrantly activate the NF-κB/NLRP3 inflammatory axis via PIEZO1, significantly elevating the secretion levels of pro-inflammatory cytokines (113). Transcriptomic analysis further confirms that under the impact of abnormal loading, the PIEZO1- Ca^2+^ signaling hub synergistically activates the PI3K-AKT and NF-κB pathways. The crosstalk between these multidimensional signals collectively drives the pathological injury and degeneration of musculoskeletal tissues (114). Furthermore, PIEZO1 mediates mechanically induced metabolic remodeling by precisely regulating arginine and proline metabolic flux, which drives the phenotypic transformation of fibroblasts into myofibroblasts and the remodeling of metabolic lineages, ultimately accelerating the progression of skin and tissue fibrosis (115, 116).

Another core site that synergistically senses signals with ion channels is focal adhesions (FAs). As dynamic complexes connecting the extracellular matrix (ECM) to the cytoskeleton, FAs and their central molecule, focal adhesion kinase (FAK), undertake the critical function of transducing physical stimuli into intracellular signals (117, 118). By integrating mechanosensitive pathways such as YAP/TAZ, RhoA/ROCK, and Piezo1, FAK facilitates extensive crosstalk between proliferation and survival signals, thereby coordinating the biological response of cells to the physical environment (117, 119). Research has further confirmed that FAK signaling plays a pivotal role in the proliferation, migration, and pro-inflammatory activation of macrophages; its overexpression is closely linked to chronic inflammation and impaired healing, whereas the inhibition of FAK can drive macrophage polarization toward the M2 phenotype (120). Preclinical evidence indicates that treatment with FAK inhibitors reduces the gene expression of inflammatory cytokines and alleviates local inflammation by decreasing the expression of IL-1β and MMP-13, thereby inhibiting cartilage degradation (121). These cartilage degradation products and pro-inflammatory mediators can diffuse into surrounding muscle groups via the local microcirculation, inducing a pro-inflammatory phenotypic remodeling of the localized skeletal muscle immune microenvironment. Concurrently, due to the loss of joint structural integrity, local biomechanical loads undergo abnormal shifting, which subsequently suppresses the FAK signaling pathway within the adjacent skeletal muscle and blocks mechanotransduction in myofibers. Evidence indicates that reactive ROS released during muscle atrophy retrogradely activate chondrocyte calcium signaling via the synovial fluid and accelerate cartilage pathogenesis (122); in turn, this exacerbated cartilage degeneration releases additional inflammatory cytokines, further deteriorating the skeletal muscle microenvironment. This cross-tissue feedback loop ultimately accentuates the mechanical and immune remodeling of skeletal muscle within the context of comorbidities.

Cellular perception of the mechanical microenvironment manifests as a rigorous cascade response extending from the cell membrane to the nucleus. At the membrane receptor level, the TRPV4 channel, acting as a broad-spectrum mechanosensor, further expands the sensing dimension of immune cells toward the mechanical microenvironment by sensing osmotic pressure and matrix deformation (123). At the level of intracellular signal transduction, the ion flux fluctuations mediated by TRPV4 synergistically activate cytoskeleton remodeling pathways such as RhoA/ROCK. This not only mediates the adhesion and chemotaxis of neutrophils but also drives the innate immune response by regulating the phagocytic activity and oxidative stress levels of macrophages (124).

Ultimately, these mechanical signals are transmitted to the intranuclear transcriptional level. Abnormal mechanical loading activates the RhoA/ROCK pathway and the translocation of NF-κB into the nucleus, initiating the transcriptional expression of pro-inflammatory genes such as IL-6 and iNOS, thereby mediating local inflammation (125). Concurrently, functional abnormalities of this pathway in primary nociceptors contribute to the development of various bone growth disorders and pathological pain by regulating neuroinflammation-related genes (123).

In summary, skeletal muscle has constructed a multidimensional mechanosensing network comprising Piezo1, TRPV4 channels, FAK complexes, and the cytoskeleton. This network precisely integrates and transforms macroscopic physical loads into intracellular biochemical signals, which not only defines the physical and mechanical characteristics of skeletal muscle but also plays an essential role in regulating immunometabolic homeostasis.

### 3.2 Dynamic loading-driven immunometabolic remodeling and muscle homeostasis maintenance

#### 3.2.1 Dynamic stress-induced redirection of energy metabolic flux and macrophage polarization

The lipotoxic microenvironment induces multidimensional tissue damage by remodeling the immunometabolic landscape of skeletal muscle. The intertwining of metabolic stress and inflammatory responses at the molecular level constitutes the core pathological basis for skeletal muscle functional decline. The core value of immune homeostasis remodeling driven by mechanical loading lies in its ability to directly antagonize the evolution of lipotoxicity-induced pathological inflammation through physical transduction signals. Research indicates a bidirectional crosstalk between lipotoxicity and the mechanical microenvironment (126, 127): lipid droplets themselves mediate intracellular mechanical stressors (126), whereas exercise can offset lipotoxic injury by improving lipid turnover (128), providing a theoretical foundation for the interventional role of mechanical loading.

Dynamic loading regulates energy metabolic remodeling through mechanotransduction, thereby influencing tissue physiological homeostasis and degenerative processes (129–131). Physiological dynamic loading can significantly alter the ratios of key energy coenzymes, such as NADP^+^/NADPH and GDP/GTP, activating glycolysis and driving energy flux into the tricarboxylic acid cycle to participate in cytoskeletal remodeling and mechanical signaling (130). Animal experiments have further confirmed that dynamic loading (such as resistance or eccentric training) can enhance oxidative enzyme activity in skeletal muscle, reshape substrate utilization preferences, and strengthen the synergistic effect between glycolysis and the tricarboxylic acid cycle, providing the energetic foundation for muscle protein synthesis (132).

Macrophages, as key regulators of the immune response, are mechanosensitive and capable of altering their lineage based on surrounding mechanical stimuli (133, 134). TRPV4 is considered a central hub mediating mechanosensing in macrophages (135–137). Research shows that pharmacological modulation of TRPV4 can induce cytoskeletal rearrangement; its agonists and antagonists can respectively drive the upregulation of CD80 (M1 type) or CD206 (M2 type) markers (135). Regarding molecular signaling pathways, 6% mechanical strain combined with TRPV4 inhibition can significantly downregulate pro-inflammatory gene expression by blocking the mitogen-activated protein kinase (MAPK) signaling pathway, thereby promoting the transition of macrophages into an anti-inflammatory state and accelerating inflammation resolution and tissue repair (136).

This molecular-level mechanotransduction exhibits a distinct strain magnitude-dependent effect within complex tissue environments. By utilizing bioreactors to simulate walking loads on bone defects under various fixation conditions, researchers found that moderate loading (5% strain) can induce the differentiation of M0 macrophages into a hybrid phenotype with highly efficient regenerative secretory functions; this simultaneously inhibits M1-type pro-inflammatory responses while promoting capillary formation. In contrast, high-level loading (35% strain) impairs the immunoregenerative response, compromising local vascularization processes and long-term mineralization efficiency (133). This indicates that mechanical stimuli exert positive immunomodulatory effects only within specific mechanical thresholds.

#### 3.2.2 AMPK–mitochondrial axis-mediated skeletal muscle quality control and functional repair

Dynamic mechanical stress is a critical biological factor in regulating the immunometabolic homeostasis of the skeletal muscle microenvironment. Research indicates that exercise intervention can synergistically promote the endogenous synthesis of pro-resolving lipid mediators while accelerating the catabolic clearance of pro-inflammatory mediators such as leukotrienes and prostaglandins, thereby timely blocking the pathological process of excessive local inflammatory infiltration (138). This dynamic evolution of the inflammatory-metabolic balance is closely associated with the improvement of macrophage mitochondrial structure and function induced by dynamic stress, and is highly dependent on the mediation of the classical AMPK signaling axis.

Mechanical loading can regulate AMPK, the central sensor of energy metabolism (139). By remodeling the metabolic flux between glycolysis and oxidative phosphorylation, AMPK can reshape the intrinsic metabolic programming of macrophages, driving their polarization toward an anti-inflammatory and reparative phenotype. This, in turn, terminates excessive immune responses and initiates tissue repair programs (140).

At the organelle level, AMPK further achieves precise mitochondrial quality control by coordinating downstream mitophagy and biogenesis pathways (141). By clearing damaged mitochondria and maintaining the homeostasis of the mitochondrial pool’s quantity and function, AMPK effectively averts metabolic disorders and oxidative stress bursts induced by the accumulation of damaged mitochondria. The imbalance of mitochondrial homeostasis is a key factor in inducing muscle protein degradation, abnormal myofiber remodeling, and subsequent muscle deterioration (142–144). By regulating mitochondria to perform pleiotropic functions, muscle functional recovery can be significantly promoted. Conversely, the exacerbation of mitochondrial dysfunction will persistently interfere with the tissue healing process (142, 144).

Appropriate dynamic mechanical stress exerts pleiotropic regulatory effects via the AMPK–mitochondrial axis, repairing mitochondrial functional defects, inhibiting the amplification of oxidative stress, and suppressing the overactivation of the cGAS-STING-mediated immune cascade. This provides essential metabolic and immunometabolic homeostasis support for myofiber repair and the maintenance of muscle function (93, 94, 139, 145–148). Animal experiments have demonstrated that in disuse muscle atrophy models established by hindlimb immobilization in C57BL/6J mice, eight weeks of resistance training increased the cross-sectional area of atrophied skeletal muscle by 23% and relative mass by 11%, while significantly improving the mice’s exercise endurance, maximum load-bearing capacity, and motor coordination. The core of this effect lies in the activation of the Piezo1/AMPK/PGC-1α signaling axis by resistance training, which promotes mitochondrial biogenesis by upregulating key protein expressions, thereby exerting biological effects that antagonize muscle atrophy (149).

Mitochondrial quality is not only the core guarantee of cellular energy metabolism but also a critical biological factor determining exercise quality and organismal endurance (150). Given that exercise performance is highly coupled with the integrity of mitochondrial ultrastructure, exercise intervention can regulate mitochondrial biogenesis, dynamic balance, and quality turnover, thereby maintaining mitochondrial functional homeostasis (151–153). The improvement of mitochondrial homeostasis driven by mechanical signals is mapped at the systemic level as exercise mode-dependent structural remodeling characteristics, endowing skeletal muscle with differentiated metabolic adaptation phenotypes.

The remodeling of skeletal muscle mitochondria by mechanical intervention modes exhibits specificity. Using multidimensional myofiber imaging and skeletal muscle biopsy techniques, Li et al. observed (n=20, young males) that systemic exercise intervention could improve mitochondrial content and volume density. Six weeks of moderate-intensity continuous training (MICT) promoted the evolution of mitochondria toward a lattice-like structure, whereas high-intensity interval training (HIIT) induced more significant longitudinal network remodeling, accompanied by the upregulation of mitochondrial fusion protein mRNA expression and the downregulation of fission protein expression (154). Furthermore, research by Botella et al. (n=28, healthy males) further revealed the underlying molecular mechanisms: a single bout of sprint interval exercise (SIE) generates severe metabolic perturbations that trigger acute mitochondrial ultrastructural and morphological disturbances, but specifically activates the mitochondrial unfolded protein response (UPRmt), the integrated stress response (ISR), and mitochondrial quality control pathways. After eight weeks of training, MICT significantly increased mitochondrial volume density, citrate synthase activity, and the expression of oxidative phosphorylation-related proteins. Conversely, while SIE did not alter mitochondrial content, it promoted the formation of respiratory chain supercomplexes by upregulating COX7A2L, thereby enhancing mitochondrial respiratory function, particularly fatty acid oxidation capacity (155).

It is noteworthy that the benefits of mechanical intervention on mitochondrial function exhibit a threshold effect. Excessive exercise training can lead to mitochondrial dysfunction and even impair glucose tolerance in healthy volunteers (156). Animal experiments have demonstrated that overtraining triggers the aberrant activation of PARP1 and its mediated protein poly-ADP-ribosylation, leading to muscular functional disturbances and impaired exercise performance. This excessive stress state blunts exercise-induced mitochondrial gains, induces muscular hyperalgesia and myofiber atrophy, and shifts the skeletal muscle gene expression profile toward patterns characteristic of myopathy and atrophy models (157).

**Figure 4:**
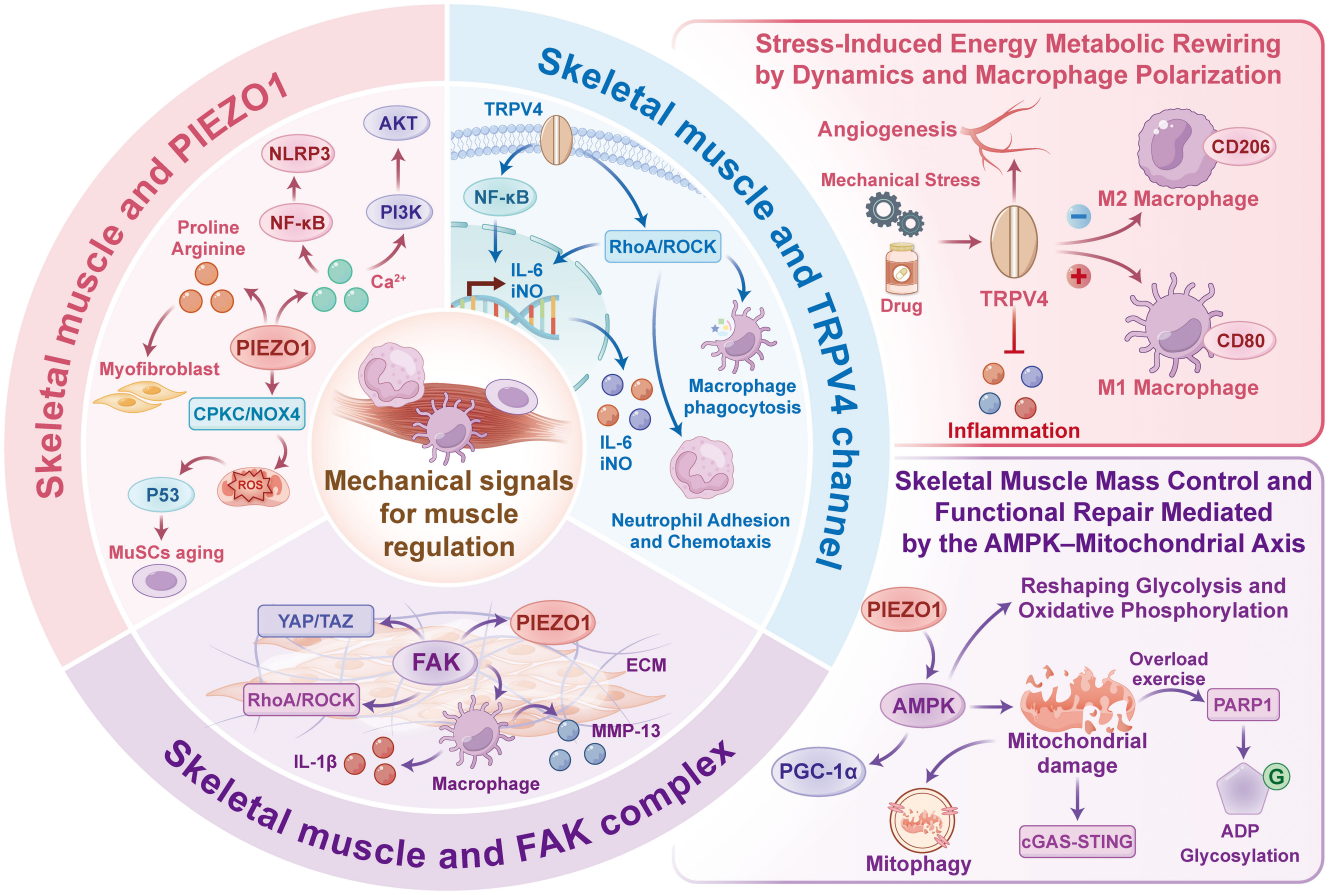
Decoding mechanotransduction in skeletal muscle: the interplay between mechanosensors, inflammatory microenvironment, and mitochondrial homeostasis.

In summary, dynamic loading precisely regulates the injury and repair processes of skeletal muscle tissue through a cascade regulatory pathway of “mechanosensing–energy metabolism–immune remodeling” (**Figure 4**). Mitochondrial quality serves as the core factor determining exercise performance, and different exercise modalities exhibit significant specificity in its remodeling. Simultaneously, the beneficial regulation of skeletal muscle and mitochondria by mechanical exercise has a clear threshold effect; overloading induces mitochondrial dysfunction and energy metabolic imbalance, leading to muscle injury and diminished exercise tolerance and performance.

From the perspective of micro-precision medicine, exercise rehabilitation operates as an integrated and targeted system. It requires the precise mapping of macro-exercise prescription elements onto the patient-specific pathological profiles—clinically aligning with the distinct mechanisms of metabolic sarcopenia or mechanical joint degeneration, while molecularly targeting the regulation of FAP adipogenic differentiation, macrophage phenotypic switching, and AMPK-mediated mitochondrial quality control. Quantitatively titrating the mechanical “dosage” to precisely ameliorate these microenvironmental biomarkers serves as the cornerstone for achieving mechanism-driven precision rehabilitation.

## 4 Conclusion

The maintenance of skeletal muscle homeostasis is essentially a highly synergistic evolutionary process of the “mechanics–metabolism–immunity” axis, with muscle mass serving as the macroscopic manifestation of their interaction. As an active interventional strategy, the core value of mechanical loading lies in its ability to block the inflammatory cascade induced by metabolic stress and to rectify pathological deviations in the immune microenvironment by modulating tissue metabolic flux. This multi-scale transformation—from mechanosensing to biological effects—not only demonstrates the metabolic adaptability of skeletal muscle under stress but also establishes mitochondrial quality control as a central hub in tissue functional reconstruction.

Crucially, the biological gains of mechanical intervention are subject to a physiological threshold; overloading triggers mitochondrial dysfunction and tissue deterioration. Therefore, the pivot of future research lies in precisely defining exercise load parameters across diverse pathological contexts. Bridging the gap from macroscopic exercise rehabilitation to microscopic precision therapy will provide the core criteria for constructing scientific and efficient clinical precision exercise prescriptions.
